# Supplementary figures and images for: MGMT ProFWise: Unlocking a New Application for Combined Feature Selection and the Rank-Based Weighting Method to Link MGMT Methylation Status to Serum Protein Expression in Patients with Glioblastoma
Source: Int J Mol Sci. 2024 Apr 6;25(7):4082. doi: 10.3390/ijms25074082 (PMC11012706; doi:10.3390/ijms25074082)

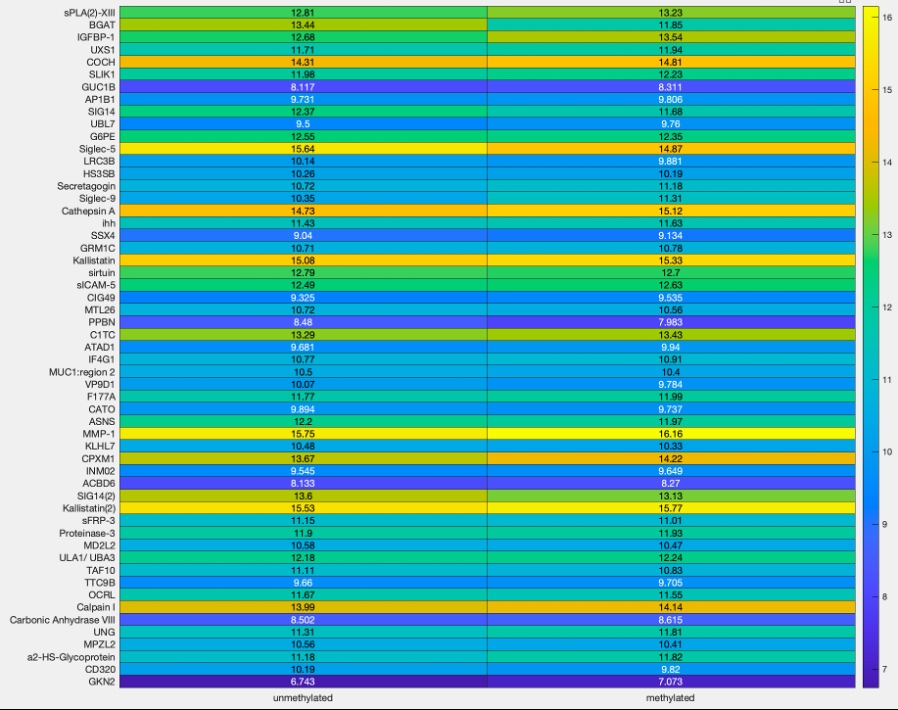

Supplement: Supplementary file 1 [file ijms-25-04082-s001.zip › Supplementary Figure1.jpg]

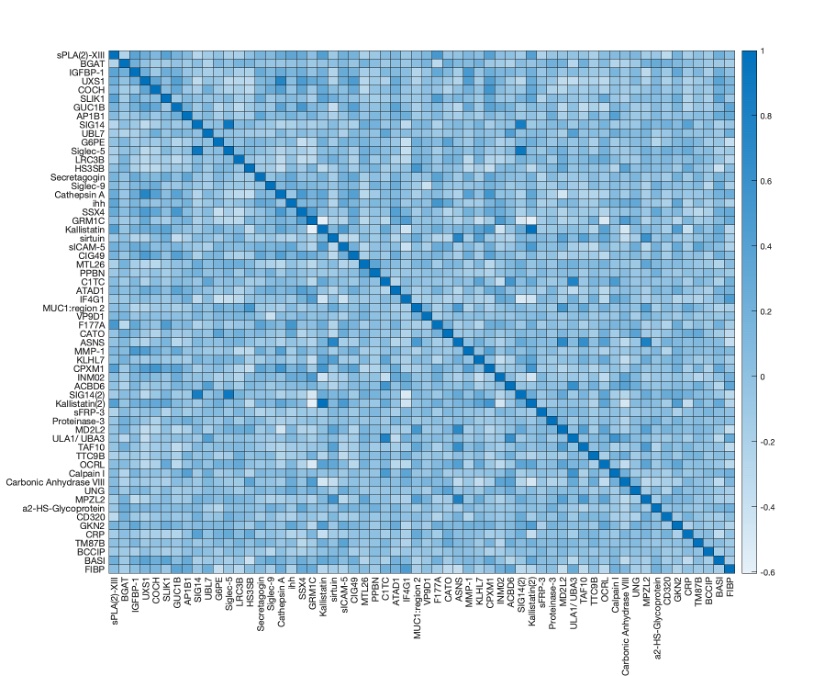

Supplement: Supplementary file 1 [file ijms-25-04082-s001.zip › Supplementary Figure2.jpg]
